# Oral cancer awareness among dentists: what is missing? A cross-sectional study

**DOI:** 10.1186/s43046-025-00290-2

**Published:** 2025-06-23

**Authors:** Dalia Ghalwash, fatheya Zahran

**Affiliations:** 1 The British University in Egypt, El Sherouk City, Egypt; 2 Cairo University, Giza, Egypt

**Keywords:** Oral cancer, Awareness, Attitude, Dentist, Practice, Egypt

## Abstract

**Introduction:**

Oral cancer (OC) is one of the major global health problems with a high incidence rate in developing countries. Early detection can improve the prognosis and survival rate of the disease.

**Aim:**

The current study evaluates dentists’ general awareness, knowledge, attitude, and practice regarding oral cancer.

**Methods:**

In the current cross-sectional study, a self-reported questionnaire was distributed to a sample of dentists in Egypt. A total of 700 dentists participated. The questionnaire included 27 questions on oral cancer knowledge, opinions, attitudes, and practices.

**Results:**

The highest awareness of risk factors concentrated around tobacco and alcohol consumption, and the most acknowledged clinical presentations were non-healing ulcers, red lesions, white lesions, and induration. The tongue was considered a high-risk site by 69% of participants, followed by the floor of the mouth and the buccal/lip mucosa. Only 37% of the participants carried out proper clinical screening for OC, while only 31% carried out routine lymph node examinations. Sixty-six percent of participants considered themselves incompetent regarding detection of OC. Ninety-two percent of participants acknowledged the important role of dentists in the early detection of oral cancer, and 99% of them thought that oral cancer awareness campaigns are needed and would be effective. Awareness was significantly associated with years of practice.

**Conclusion:**

Awareness regarding OC among the Egyptian dentists participating in the current survey showed definitive defects. Hence, efforts to raise awareness of OC among dental practitioners are an important factor in improving/early detection of OC, with the resultant increase in survival rate and decrease in morbidity. This can be reached only through more solid undergraduate syllabi and training as well as workshops and campaigns.

## Introduction

Oral cancer (OC) is one of the major global health problems, occupying the eighth rank among different known types of cancers [1]. The estimated number of new cases exceeds 450,000 annually worldwide, with a higher prevalence in developing countries [2]. It is accountable for about 3.7% of cancer-related cases globally [3]. According to the latest WHO data published in 2020, OC deaths in Egypt reached 0.15% of total deaths. The age-adjusted death rate of OC is 1.16 per 100,000 of the population, which ranks Egypt #170 in the world [4]. Above 90% of all oral cavity cancers are oral squamous cell carcinoma (OSCC) [3, 5]. The higher morbidity and mortality in oral cancer are mainly attributed to the delay in diagnosis rather than its aggressiveness. The unfavorable five-year survival rate of oral cancer is still only 50% but can be improved to 80% if the lesion is diagnosed at an early stage [5].

In some patients, oral cancer is preceded by an oral potentially malignant disorder (OPMD). A recent analysis published by our research team showed that 10.19% of cases with oral lichen planus, an OPMD, could show dysplastic changes [6]. Diagnosing lesions in this stage might reduce the frequency of oral malignancy, increase patients’ survival rate, and improve quality of life [7–9]. A recent scoping review concluded the growing prevalence of head and neck cancer in Egypt, which indicates the need for more research focusing on etiologies, risk factors, treatment, and prevention [10].

As the majority of oral cancers are associated with lifestyle risk factors, including smoking, alcohol consumption, betel nut chewing, poor diet, aging, and family history of cancer [11], enhancing the awareness of how important avoiding and monitoring these factors is imperative in the primary prevention strategies for oral cancer.

Despite the easy accessibility of the oral cavity for clinical examination and routine screening, which would facilitate early detection of malignant changes, oral cancer remains a highly lethal disease and one of the most debilitating and disfiguring of all malignancies [7, 11].

Patients with oral lesions often initially present to general dental practitioners, putting them in a unique place where they can discover oral cancer in its initial stages [12–14]. Consequently, oral cancer prevention principally relies on general dental practitioners, which highlights the importance of their level of knowledge and awareness regarding oral cancer signs and symptoms in addition to its risk factors [14]. Another important aspect of the prevention of oral cancer is encouraging dentists to routinely inspect the oral mucosa of every patient, especially those at higher risk such as smokers or alcohol consumers [15].

Several studies have assessed dental professionals’ knowledge, attitudes, and practice about oral cancer in different places all over the world, but there is still a need for more similar studies in different countries [7, 16, 17]. To our knowledge, the number of studies regarding dentists’ awareness and attitude towards oral cancer is very limited in Egypt. Therefore, the current study is designed to evaluate dentists’ general awareness, knowledge, attitude, and practice about oral cancer among a sample of Egyptian dentists.

## Methods

The present observational cross-sectional study was carried out to assess oral cancer awareness among dentists employing a self-developed anonymous questionnaire. The questionnaire is comprised of twenty-seven questions that were divided into four sections. The first section included 6 questions related to personal information, and the first one concerned the approval to participate in the questionnaire. The second section included 7 questions regarding general awareness and knowledge, 6 questions regarding general attitude, and 8 on general practice.

### Tool reliability and internal consistency of the questionnaire

An internal pilot study was carried out before carrying out a full-scale study to assess tool reliability and validity on 20 participants who were excluded from the full-scale study. The knowledge domain was composed of 7 questions with a Cronbach alpha coefficient assessing internal consistency of 0.978, the attitude domain was composed of 6 questions with a Cronbach alpha coefficient assessing internal consistency of 0.982, and the practice domain was composed of 8 questions with a Cronbach alpha coefficient assessing internal consistency of 0.971. The internal consistency of the 3 domains illustrates an excellent level. The test–retest was done to assess the reliability and demonstrates that interclass correlation for questions ranged from 0.789 to 0.895, indicating excellent agreement.

The questionnaire was elaborated on a Google form and sent via e-mail and messages to more than 1000 graduate dentists in all fields of dental practice, either governmental, private, or educational institutions in different Egyptian governorates. When we received the complete responses of 700 dentists, which represents the calculated sample size for the study, the data was then transferred to an Excel sheet, and descriptive statistical analysis was conducted.

### Sample size calculation

Based on previous research regarding dentists’ awareness of the diagnosis of oral cancer in Upper Egypt [18], the minimal sample to achieve a statistically significant result was calculated as 700. The odds ratio is 1.5; by fixing alpha at 0.05 and beta at 0.8.

Ethical approval of the study was acquired from the Research Ethics Committee of the Faculty of Dentistry at the British University in Egypt with approval number 24–033.

### Statistical analysis and data interpretation

Data analysis was performed by SPSS software, version 26 (SPSS Inc., PASW Statistics for Windows version 26. Chicago: SPSS Inc.). Qualitative data were described using numbers and percentages. The significance of the obtained results was judged at the ≤ 0.05 level. Monte Carlo tests were used to compare qualitative data between groups as appropriate.

## Results

**Fig. 1:**
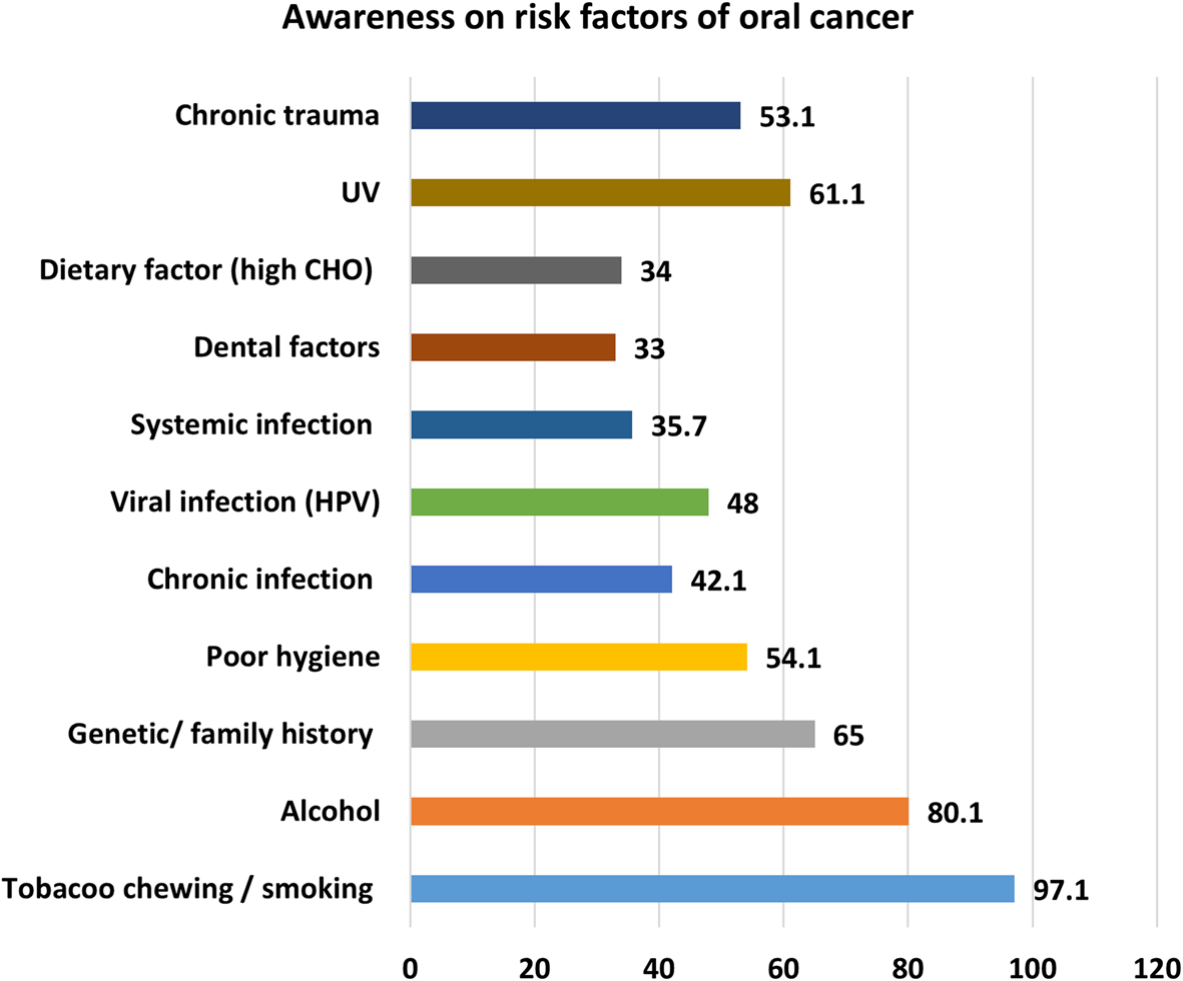
Awareness of oral cancer risk factors

A total of 700 dentists participated in the current cross-sectional study. The demographic characteristics of the participants are presented in Table 1. Among the 700 participants, 329 (47.0%) were general practitioners in public dental practice, 231 (33.0%) were general practitioners in private dental practice, and 140 (20%) worked in educational institutions. Data concerning the general awareness and knowledge among studied participants are presented in Table 2. Awareness of the participants concerning oral cancer risk factors is shown in Fig. 1. The most important remark in the figure is that the highest awareness of risk factors is concentrated around tobacco and alcohol consumption, followed by UV exposure and genetic factors, whereas many other equally or even more important factors were not acknowledged by a huge percentage of participants.

**Table 1:** Demographic characteristics of the studied participants

		***N*** = 700	%
Age	22–29 years	315	45.0
	30–39 years	161	23.0
	40–49 years	161	23.0
	50–59 years	49	7.0
	60 years and older	14	2.0
Gender	Female	427	61.0
	Male	273	39.0
Educational level	Bachelor’s degree	504	72.0
	Master’s degree	112	16.0
	PhD	84	12.0
Years of practical experience	Less than 5 years	77	11.0
	5–10 years	119	17.0
	11–15 years	266	38.0
	More than 15 years	238	34.0

**Table 2:** General awareness and knowledge among studied participants

General awareness and knowledge	*N*	%
Is oral cancer preventable?	441	63.0
Is oral cancer treatable?	406	58.0
Does the risk of getting oral cancer increase with age?	679	97.0
Which of the following are risk factors of oral cancer?		
Tobacco chewing/smoking	680	97.1
Alcohol	561	80.1
Genetic/family history	455	65.0
Poor hygiene	379	54.1
Chronic infection	295	42.1
Viral infection (HPV)	336	48.0
Systemic infection	250	35.7
Dental factors	231	33.0
Dietary factor (high CHO)	238	34.0
UV	428	61.1
Chronic trauma	372	53.1
Which of the following is a possible clinical presentation of oral cancer?		
Bleeding	196	28.0
Paresthesia	119	17.0
Necrosis	175	24.0
Pain	168	24.0
Change in color	351	50.1
Non-healing ulcer	595	85.0
Hyperkeratotic mucosa	281	40.1
Lymphadenopathy	359	51.3
Induration	497	71.0
Swelling	329	47.0
Red lesion	589	84.1
White lesion	568	81.1
Which sites in the oral cavity are at higher risk of developing oral cancer?		
Tongue	483	69.0
The floor of the mouth	434	62.0
Buccal/labial mucosa	434	62.0
Palate	217	31.0
Posterior part	336	48.0
All sites	210	30.0
How far is your knowledge concerning the prevention and detection of oral cancer?		
Adequately informed	406	58.0
Poorly informed	175	25.0
Well informed	119	17.0

**Fig. 2:**
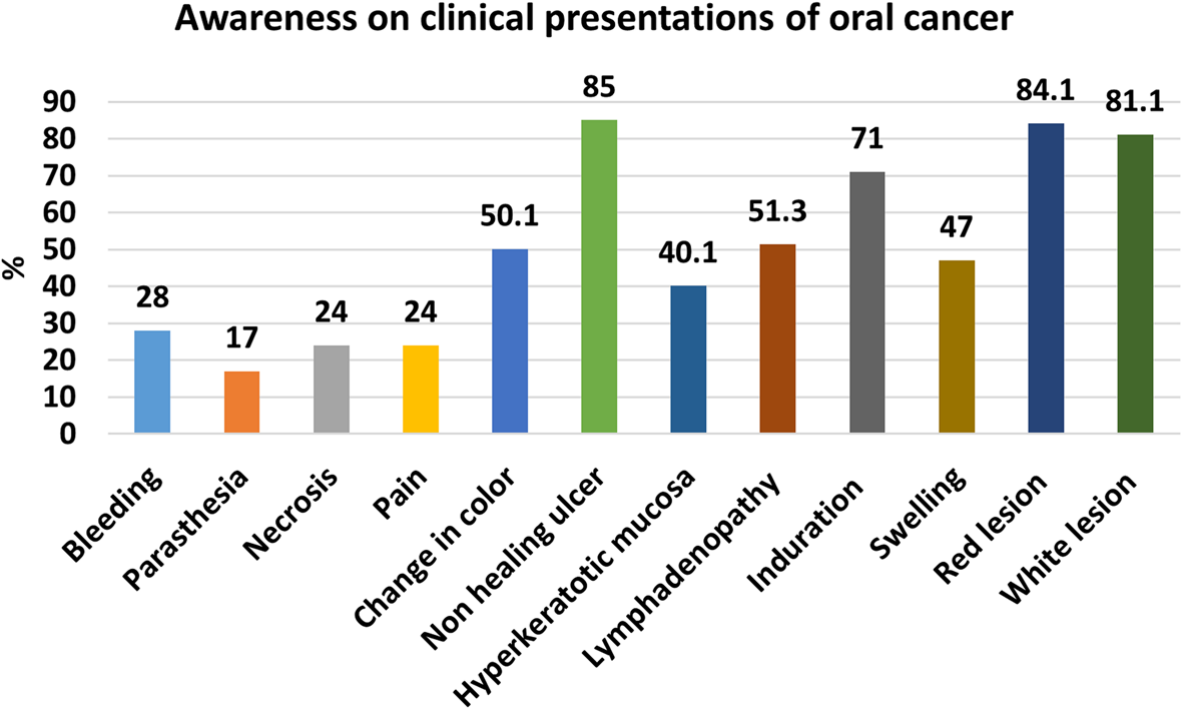
Awareness of clinical presentations of oral cancer

**Fig. 3:**
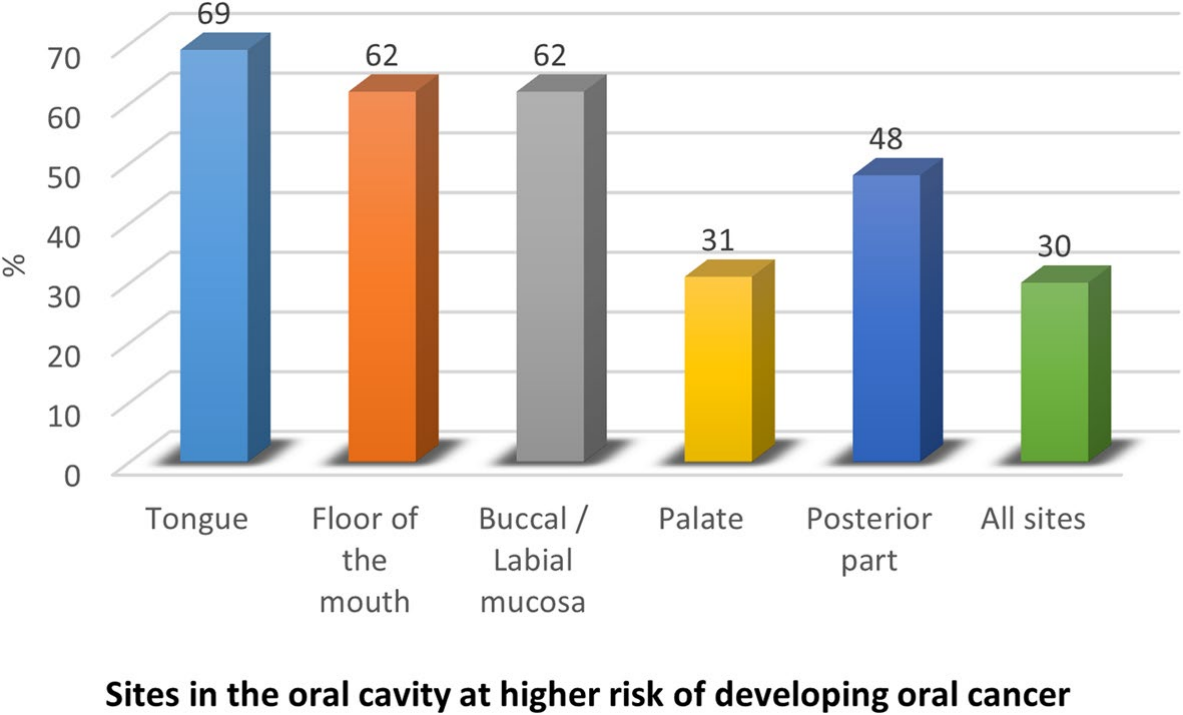
Participants’ knowledge concerning high-risk sites of oral cancer

Awareness of the clinical presentations of oral cancer is demonstrated in Fig. 2 showing that the most acknowledged clinical presentations by the participants were non-healing ulcers, red lesions, white lesions, and induration. Participants’ knowledge concerning sites at high risk of developing oral cancer is presented in Fig. 3. Data regarding the general attitude among studied participants are displayed in Table 3.

**Table 3:** General attitude among studied participants

General attitude	*N*	%
Do you think you are competent to detect oral cancer?		
No	462	66.0
Yes	238	34.0
Will you deny treatment to patients with oral cancer?	56	8.0
Would you get yourself and advise your friends and family to get screened for oral cancer?	637	91.0
Do you feel that oral cancer awareness campaigns are needed and would be effective?	693	99.0
Do you think dentists have an important role in the early detection of signs and symptoms of oral cancer?	644	92.0
Do you think dentists should advise patients to avoid the risk factors of oral cancer?	574	82.0

Concerning the general practice among the studied participants, data are presented in Table 4. Strikingly, only 37% of participants routinely examine the patient’s oral cavity for signs of OC, only 31% routinely examine lymph nodes, and only 30.5% know the clinical criteria of malignancy in lymph nodes.

**Table 4:** General practice among studied participants

General practice	*N*	%
Do you record tobacco and alcohol use in your personal history?	546	78.0
Do you routinely examine the patient’s oral cavity for signs of oral cancer?	259	37.0
Do you routinely examine the head and neck lymph nodes of your patients?	217	31.0
What signs make you suspicious of cancer when you examine head and neck lymph nodes?	*N* = 623	
LNs are fixed, painful, and hard	314	50.4
LNs are fixed, painless, and hard	190	30.5
LNs are movable, painless, and hard	70	11.2
LNs are movable, painless, and soft	49	7.9
Have you ever diagnosed a case of a potentially malignant lesion?	203	29.0
Have you ever detected a case of oral cancer?	105	15.0
Can you obtain a biopsy specimen from a suspicious oral lesion?	84	12.0
Which specialty would you refer cases with suspicious oral lesions to?		
Oncology	126	18.0
Oral Medicine	413	59.0
Oral Surgery	161	23.0

Table 5 demonstrates the association between years of experience and awareness, attitude, and practice.

**Table 5:** Association between years of experience and awareness, attitude, and practice

	Years of practical experience				Test of significance
	< 5 *N* = 266(%)	5–10 *N* = 119(%)	11–15 *N* = 77(%)	> 15 *N* = 238(%)	
General awareness and knowledge					
Is oral cancer preventable?	147 (55.3)	84(70.6)	49(63.6)	161(67.6)	Mc = 11.99 *P* = 0.007*
Is oral cancer treatable?	126(47.4)	49(41.2)	63(81.8)	168(70.6)	Mc = 59.58 P < 0.001*
Does the risk of getting oral cancer increase with age?	259(97.4)	112(94.1)	77(100)	231(97.1)	Mc = 5.91 *P* = 0.116
Do you have sufficient knowledge concerning the prevention and detection of oral cancer?					
Adequately informed	161(60.5)	49(41.2)	35(45.5)	161(67.6)	Mc = 51.28*P* < 0.001*
Poorly informed	70(26.3)	49(41.2)	14(18.2)	42(17.6)	
Well informed	35(13.2)	21(17.6)	28(36.4)	35(14.7)	
General attitude					
Do you think you are competent to detect oral cancer?	56(21.1)	42(35.3)	42(54.5)	98(41.2)	Mc = 39.91 *P* < 0.001*
Will you deny treatment to patients with oral cancer?	21(7.9)	7(5.9)	0(0.0)	28(11.8)	Mc = 12.01 *P* = 0.007*
Would you get yourself and advise your friends and family to get screened for oral cancer?	252(94.7)	112(94.1)	63(81.8)	210(88.2)	Mc = 16.09 *P* = 0.001*
Do you feel that oral cancer awareness campaigns are needed and would be effective?	266 (100)	119(100)	77(100)	231(97.1)	Mc = 13.73 *P* = 0.003*
Do you think dentists have an important role in early detection of signs and symptoms of oral cancer	238 (89.5)	112(94.1)	70(90.9)	224(94.1)	Mc = 4.61 *P* = 0.203
Do you think dentists should advise patients to avoid the risk factors of oral cancer?	210(78.9)	77(64.7)	63(81.8)	224(94.1)	Mc = 49.47 *P* < 0.001*
General practice					
Do you record tobacco and alcohol use in your personal history?	175(65.8)	105(88.2)	70(90.9)	196(82.4)	Mc = 40.48 *P* < 0.001*
Do you routinely examine the patient’s oral cavity for signs of oral cancer?	49(18.4)	28(23.5)	49(63.6)	133(55.9)	Mc = 108.49 *P* < 0.001*
Do you routinely examine the head and neck lymph nodes of your patients?	42(15.8)	35(29.4)	28(36.4)	112(47.1)	Mc = 58.64 *P* < 0.001*
Have you ever diagnosed a case of a potentially malignant lesion?	14(5.3)	14(11.8)	42(54.5)	133(55.9)	Mc = 197.89 *P* < 0.001*
Have you ever detected a case of oral cancer?	0	0	7(9.1)	98(41.2)	Mc = 197.96 *p* < 0.001*
Can you obtain a biopsy specimen from a suspicious oral lesion?	0	0	14(18.2)	70(29.4)	Mc = 123.61 *P* < 0.001*

*MC*, Monte Carlo test

^*^Statistically significant

## Discussion

Although oral cancer is one of the diseases that can be detected in the early stage through routine clinical examination of the accessible oral cavity, most of the cases are recognized in the late stages of the disease [14]. Early detection of oral cancer would result in more efficient and timely management and is considered a key factor in reducing the mortality and morbidity of oral cancer. Dentists can play a major role in implementing oral cancer detection and prevention measures that could improve the survival rates of oral cancer patients [19]. However, inadequate knowledge regarding oral cancer has been widely documented in current research among general dental practitioners from developed and developing countries [16]. Moreover, a lack of awareness of oral cancer risk and clinical signs may prohibit dentists from delivering preventive advice [19].

Thus, the current investigation was intended to assess the level of awareness, knowledge, attitude, and practice towards oral cancer among Egyptian dentists. An anonymous questionnaire was used to encourage the participants to express their actual behavior regarding the different questionnaire items. This had an impact on the reduction of information bias.

In the current study, data concerning awareness of oral cancer risk factors revealed that 97% of participants believed that the risk of getting oral cancer increases with increasing age, Additionally, tobacco chewing/smoking, alcohol consumption, and family history were the commonly recognized risk factors of oral cancer by 97.1%, 80.1%, and 65% of participants respectively. Tobacco use is the most important risk factor for oral cancer [20], and its identification by the majority of participants points out that their knowledge is consistent with the contemporary understanding of the etiology of oral potentially malignant and malignant lesions reported by several studies [11, 21, 22]. However, other factors such as viral infections, dietary factors, bad oral hygiene, and dental and systemic factors were not acknowledged beyond one-third of the participants. Thus, although 82% of participants recommended that dentists should warn their patients to avoid OC risk factors, this percentage is not as impressive as it would seem because a smaller percentage of dentists appeared to be aware of the whole range of risk factors to give their patients comprehensive advice. It is well-established that oral cancer is largely related to lifestyle, and as healthcare providers, dental practitioners should be well aware of these factors and play a central role in providing information about the benefits of changing lifestyle habits [7, 11, 23]. Also, defining the patients at risk is important as an alert for more careful examination and follow-up. Awareness of the clinical presentations of oral cancer showed that the most acknowledged clinical presentations by the participants were non-healing ulcers (85%), red lesions (84.1%), white lesions (81,1%), and induration (71%). Such results were encouraging as they conformed to previous literature [24].

Data regarding the general attitude among studied participants displayed that 92% of them acknowledged the important role of dentists in the early detection of oral cancer, however, only one-third of participants declared their routine examination of lymph nodes. Also, a similar percentage reported routine screening of the oral mucosa for signs of OC. These two responses look contradictory. Although most of the participants declare the importance of the dentist’s role in the early detection of oral cancer, we find a good percentage refraining from screening and lymph node examination. This highlights the practitioners’ lack of ability or motivation to carry out the screening [25]. Lack of training and lack of confidence could be the underlying causes. Unfortunately, these shortages in knowledge and practice are a real threat to the prevention and early detection of the disease and subsequently reducing its burden, considering that the majority of the global burden of oral cancer lies in the developing world [2, 3]. Similar findings have been reported in previous research [12, 26, 27]. Therefore, educational strategies should be aimed at providing current information on oral examinations, diagnostic techniques, and conditions associated with oral cancer. The proof for such an assumption came from the present results, where 25% of participants believed they were poorly informed about the prevention and detection of oral cancer, 58% of participants believed they were adequately informed, and only 17% thought they were well informed. In addition, 99% of participants thought that oral cancer awareness campaigns were needed and would be effective.

Sixty-nine percent of participants considered the tongue a high-risk site, followed by the floor of the mouth and the buccal/lip mucosa. This is in accordance with most available literature [28–30].

The relation between years of practice and OC awareness revealed significant differences between the categorized participants, mostly in favor of older groups. This was in some instances, logical as the questions around previous detection of oral potentially malignant and malignant lesions and taking biopsy, all of which can be more experienced with a longer duration of practice. However, it was so discouraging when the youngest group failed to practice oral screening, lymph node examination, and other skills that they have been recently taught as they have recently graduated, and nowadays, much care is put into inserting OC-related information and practice within undergraduate curricula. One of the important points showing no significant difference between groups with varying periods of practice was that dentists have an important role in the early detection of signs and symptoms of oral cancer, where all groups showed high percentages of approval, thus confirming a general consensus. Dental practitioners are in an ideal position to help people quit smoking because they are among the few healthcare professionals who routinely see “healthy individuals” [12]. Saleh et al. [20] found that many dentists were uncomfortable discussing OC risk habits with their patients and commented that this scenario was not unique to Malaysia [20]. Studies in the United Kingdom and European Union reported that most dentists do not determine their patients’ tobacco habits and provide cessation counseling [27–29], whereas, in the United Kingdom, only 30% of dentists provide brief cessation advice to their patients. A similar situation was reported in the United States of America, where 60% of dentists do not routinely advise tobacco users to quit [31].

Previous research presented ideas regarding undergraduate teaching of screening for OC and oral potentially malignant diseases (OPMDs), aiming at increasing awareness and skills among graduated dental practitioners. Great scenarios were discussed, including problem-based learning, clinical case presentations, and virtual learning (which can compensate for the limited number of cases), besides the didactic part [32]. However, all suggestions were focused on Oral Pathology and Oral Medicine curricula. Although the described methods of teaching with all details included seemed outstanding [32], making it part of Oral Pathology and Oral Medicine curricula only makes it vulnerable to be considered by students as just a chapter in these curricula to be studied for the exams and then totally forgotten. Whether in Cairo University or the British University in Cairo, as well as in most dental schools in other Universities in Egypt, Oral Medicine curricula contain a limited part dealing with early detection of oral cancer. There is one lecture dedicated to the topic, including all techniques used up to date, and then in the clinics, only 20% of the sessions are dedicated to the skills required for screening for oral cancer, ending in a clinical assessment. Oral Pathology curricula have single chapters dedicated to oral cancer, of course, without any clinical application and without integration with Oral Medicine or Oral Radiology in that regard.

Actually, we need the practice of screening for OC and OPMDs to reach the level of skills that dentists acquire in general dental specialties, such as conservative and prosthodontics dentistry and exodontia. This necessitates a vertical distribution throughout all academic years with a merge from a preclinical to a clinical level, followed by scheduled practice during the internship year. Hence, we need an early start, perhaps with the curricula of general pathology and general surgery, where the process of carcinogenesis can be taught with reference to both its histopathology and clinical presentation through all its different stages till it reaches its full-blown picture of malignancy with its diverse forms and levels of differentiation. Then comes the part to be played by Oral Pathology and Oral Medicine staff. However, these two specialties are not to do it individually; it should take the form of integrated sessions, together with Oral and Maxillofacial Radiology and Oral and Maxillofacial Surgery staff. Throughout these sessions, risk factors, methods of screening, investigations, diagnosis, case referral protocols, and every single thing related to OC and OPMDs should be taught and supplemented with clinical practice [33]. Then comes the role of public health, where curricula should include an integral part related to tobacco cessation strategies, which should be practiced by students. Also, methods related to persuading patients’ persuasion into more healthy lifestyles and the evasion of all potential risk factors should be included. Students should be properly instructed that patient education concerning risk factors is one of the main duties of a dental practitioner. The internship year should include one whole round for practicing all that is learned before graduation, equivalent to practicing other dental specialties. After that, continuous education programs should always include workshops for the early detection of OC and OPMDs and implementing strategies to develop motivation and self-learning [34]. There should be an assessment for skills and competencies gained in this context held simultaneously during practice license exams for new graduates. An audit can even be added at the time of license renewal. This should be generalized, not only applied by some institutes and leaving out the rest. Ramirez-Amador et al. have even recommended the exchange of ideas and experiences among different countries to reach a unified, effective, and internationally accepted curriculum aiming at solid knowledge, awareness, and skills related to a very important competency, which is oral cancer early detection and dealing with any carcinogenic process in the oral cavity [32].

The primary limitation of this study is the study population, which was comprised mainly of dentists from Cairo. A future study should expand to include other dental schools and other areas of Egypt, as well as other oral health care providers in private clinics for a more comprehensive sample. Another possible limitation is the tendency of dentists to provide acceptable answers, which may differ from what they actually do, and this might bias the outcome. However, the anonymous nature of the questionnaire should have minimized this type of information error.

## Conclusion

It could be concluded that awareness regarding OC among the Egyptian dentists participating in the current survey has definitive defects. Above all, there seems to be a deficiency in the awareness of many well-documented risk factors and clinical signs of OC. Furthermore, only one third of the participants carry out proper clinical screening for oral mucosal lesions or lymph node examination among their patients. In contrast, two thirds of participants consider themselves incompetent regarding detection of OC. Hence, efforts to raise awareness of OC among dental practitioners are an important factor in improving early detection of OC, with the resultant increase in survival rate and decrease in morbidity.

## Recommendations

Raising awareness of oral cancer among dental practitioners can be achieved through more continuing education programs on risk factors and oral cancer diagnosis to train dentists. Oral cancer screening should be a routine procedure for high-risk patients at the primary oral health care centers as well as in the private sector.

In dental education, more training and exposure to oral cancer screening is urgently needed, and the competency for providing risk habit cessation advice should be added to undergraduate dental curricula. It should be done with a multidisciplinary approach. Furthermore, currently licensed practitioners should have mandatory continuing education courses on oral cancer. This could be an important approach to achieving the goals of OC detection and early treatment. Also, routine checkups and prophylaxis should have more emphasis, and information about oral cancer should be delivered to both patients and dental professionals, whether general or specialized.

## Data Availability

No datasets were generated or analysed during the current study.
